# Highly Iterated Palindromic Sequences (HIPs) and Their Relationship to DNA Methyltransferases

**DOI:** 10.3390/life5010921

**Published:** 2015-03-17

**Authors:** Jeff Elhai

**Affiliations:** Center for the Study of Biological Complexity, Virginia Commonwealth University, Richmond, VA 23284, USA; E-Mail: ElhaiJ@vcu.edu; Tel.: +1-804-828-0794; Fax: +1-804-828-0503

**Keywords:** repeated sequence, methyltransferase, HIP1, cyanobacteria, restriction/modification

## Abstract

The sequence GCGATCGC (Highly Iterated Palindrome, HIP1) is commonly found in high frequency in cyanobacterial genomes. An important clue to its function may be the presence of two orphan DNA methyltransferases that recognize internal sequences GATC and CGATCG. An examination of genomes from 97 cyanobacteria, both free-living and obligate symbionts, showed that there are exceptional cases in which HIP1 is at a low frequency or nearly absent. In some of these cases, it appears to have been replaced by a different GC-rich palindromic sequence, alternate HIPs. When HIP1 is at a high frequency, GATC- and CGATCG-specific methyltransferases are generally present in the genome. When an alternate HIP is at high frequency, a methyltransferase specific for that sequence is present. The pattern of 1-nt deviations from HIP1 sequences is biased towards the first and last nucleotides, *i.e.*, those distinguish CGATCG from HIP1. Taken together, the results point to a role of DNA methylation in the creation or functioning of HIP sites. A model is presented that postulates the existence of a G^me^C-dependent mismatch repair system whose activity creates and maintains HIP sequences.

## 1. Introduction

Many bacterial genomes exhibit particular oligomeric DNA sequences, typically of length 5 to 10 nt, at a far higher frequency than would be expected by chance [1,2]. One class of such sequences are Chi (Crossover Hotspot Initiator) sites, required for RecBCD-mediated recombination [1]. A second class consists of oligomers recognized by DNA uptake systems in naturally transformable bacteria [2].

A third class was described by Robinson *et al.* (1995) [3], HIP1 (Highly Iterated Palindrome) sites found overrepresented in many cyanobacteria [3,4]. The sequence, GCGATCGC, is the same in all described instances [4], although a larger sequence, GGCGATCGCC, has been recognized in two cyanobacteria [2,5]. Little is known about the biological significance of the sequence, but there is some indication that it may function in site-specific recombination in *Synechococcus* PCC 7002 [5] and (at a much lower efficiency) illegitimate recombination between cloned sequences in *Synechococcus* PCC 7942 and *Escherichia coli* [6].

A possible connection between HIP1 sequences and two DNA methyltransferases (MTases) has been previously noted [4,7,8]. The internal four nucleotides, GATC, is methylated by the MTase DmtA, found in *Anabaena* PCC 7120, an essential protein under standard laboratory conditions [7]. The apparent ubiquity [9] of this enzyme amongst cyanobacteria (excluding the picocyanobacteria) inspired the suggestion that it may be related to the function of the equally ubiquitous HIP1 [4]. A second MTase, specific for CGATCG and called M.Ssp6803I in *Synechocystis* PCC 6803 [8] and DmtC in *Anabaena* PCC 7120 [7], also lies within HIP1, and mutant *Synechocystis* lacking this MTase were unable to grow under conditions favoring rapid growth [8]. MTases recognizing CGATCG are the second most common amongst cyanobacteria, behind only those recognizing GATC [9].

Exceptions to a rule may be useful in understanding the basis of that rule, and exceptions exist to the rule that cyanobacteria (apart from the picocyanobacteria) have overrepresented HIP1 sequences. There is considerable variation in the frequency of HIP1 sequences, even after accounting for nucleotide abundance [4], and the apparent absence of HIP1 sequences in *Calothrix* D253 [3] led Robinson *et al.* (1998) [6] to conjecture that the presence of the sequences may be polyphyletic.

The present work was initiated to examine these exceptional cases more closely and to see whether the presence of the DmtC MTase may provide clues as to the function or maintenance of HIP1 sequences.

## 2. Results

### 2.1. Genomes Considered in This Study

**Figure 1:**
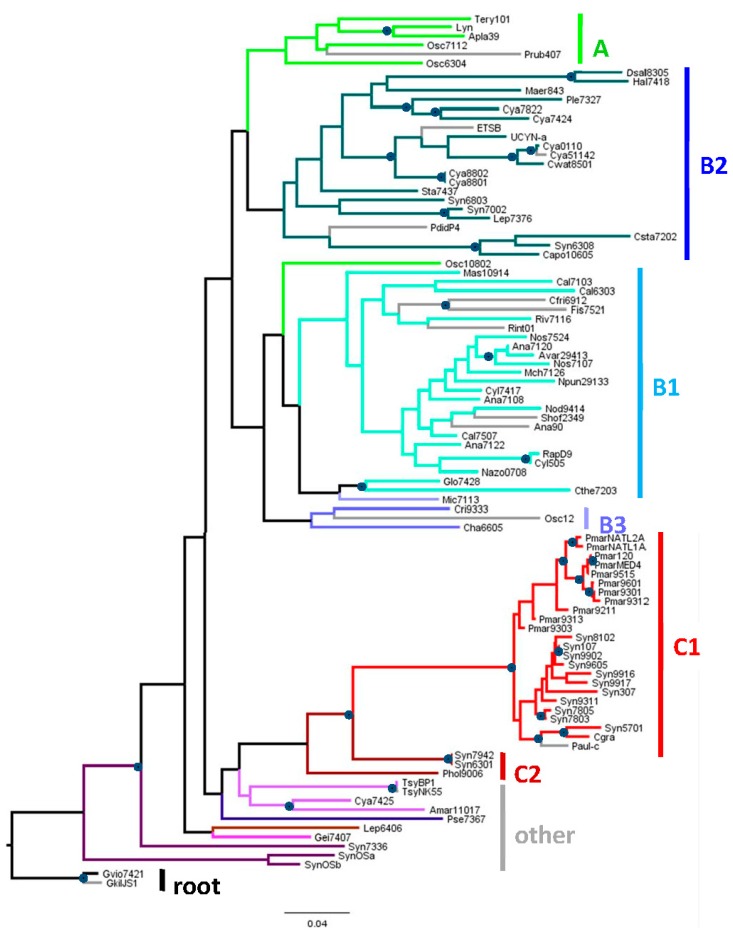
Phylogenetic tree of 16S rDNA from organisms used in this study. The maximum likelihood tree was based on complete 16S rDNA sequences and rooted by *Gloeobacter*, in accordance with previous trees that used various eubacterial sequences as outgroups [11,12]. Organism abbreviations are explained in Supplemental Table S1. The categories of organisms, Groups A through **C** are taken from Shih *et al.* (2013) [11], Figure 1A. Branches are color-coded to facilitate identification with the categories shown to the right of the tree. Gray branches lead to organisms not in the set used by Shih *et al.* (2013). Circles at nodes indicate those that are supported by at least 70 of 100 bootstrap trials. *Leptolyngbya* Heron Island J was omitted from the construction of the tree because only 71% of its 16S rRNA sequence is known. However, by inspection of the aligned sequences, it is very close to *Leptolyngbya* PCC 6406. The bar represents the horizontal distance corresponding to 0.05 mutations per aligned nucleotide.

The genomes considered in this study, taken from those currently in the CyanoBIKE database [10], are shown in a phylogenetic tree based on 16S rRNA sequences (Figure 1), with additional information provided in Supplemental Table S1. The tree adds 14 genomes to those in the 16S tree of Shih *et al.* (2013, their Figure S2) [11]. The two trees are completely concordant, except for a discrepancy in the placement of *Prochlorococcus marinus* CCMP 1375 (ss120), and both suffer from low bootstrap support, as compared to the tree constructed by Shih *et al.* [11] by the alignment of conserved protein. The differences between the 16S and protein trees (seen in Figure 1 as branches with anomalous colors) are minor and unimportant for this study).

I will refer to major groups of cyanobacteria according to the nomenclature of Shih *et al.* (2013) [11], since the traditional morphological divisions of cyanobacteria [13] do not accord well with phylogenies based on genomic sequences. Group A includes many but not all of the old Section III (filamentous cyanobacteria), Group B1 contains cyanobacteria of Section IV (heterocyst-forming) and two (*Gloeocapsa* PCC 7428 and *Chroococcidiopsis* PCC 7203) from other subsections. Group B2 consists mostly of cyanobacteria in Section I and Section II (unicellular strains that divide by simple division or budding or by multiple fission, respectively). Group C1 consist of the picocyanobacteria *Prochlorococcus* and some *Synechococcus*. The other groups contain mostly high GC% cyanobacteria from Section I and Section III.

Four of the genomes come from cyanobacteria that were taken from apparently obligate symbiotic associations: *Nostoc azollae* 0708 [14] (Group B1) associated with the fern *Azolla filiculoides*, *Richelia intracellularis* HH01 [15] (Group B1) with the diatom *Hemiaulus hauckii*, UCYN-A (Group B2) with marine photosynthetic picoeukaryotes [16], and *Prochloron didemni* P4 (Group B2) with the seq squirt *Lissoclinum patella* [17]. There are also two genomes from cyanobacterially derived organelles: the spherical body or nitrosome from the diatom *Epithemia turgida* [18] (EtSB; Group B2) and the chromophore from the amoeboid *Paulinella chromatophora* [19] (Group C1).

Unfortunately, the genome sequence of the anomalous strain of *Calothrix* considered by Robinson *et al.* (1995) [3] is not available—only the 48 small sequences they deposited are present in Genbank. These sequences were used to find the closest available genome, *Calothrix* PCC 7103 (Table 1).

**Table 1:** Match of *Calothrix* D253 DNA fragments to other *Calothrix* strains. All 48 DNA fragments (totaling 19,301 nt) from *Calothrix* D253 were Blasted against each available genome of *Calothrix*. A match was defined as any hit with an *e*-value better than 10^−3^. Eight fragments (totaling 3162 nt) found matches in all three strains. An unweighted average was calculated from the %ID values of each match for each strain.

Organism	% D253 Fragments Matched	% ID in Matches to Common Fragment
*Calothrix* PCC 6303	29%	86%
*Calothrix* PCC 7103	79%	94%
*Calothrix* PCC 7507	27%	83%

### 2.2. Frequencies of HIP1 Sequences

**Figure 2:**
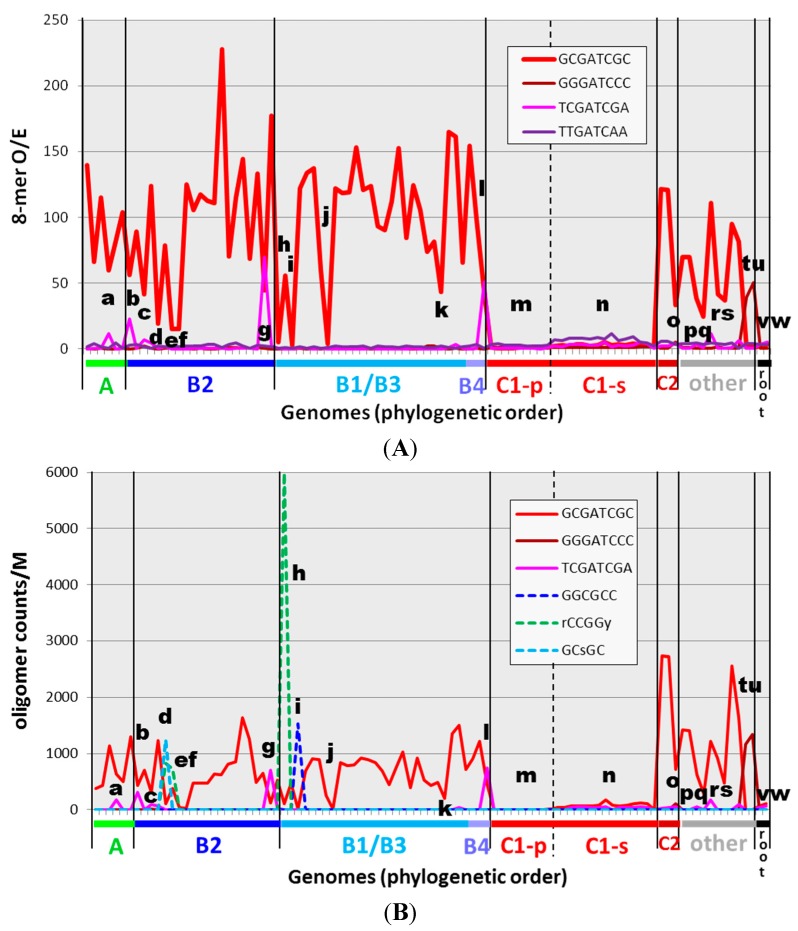
Occurrences of HIP1 and other oligomers in cyanobacterial genomes. The O/E ratio (observed/expected counts) (**A**) and filtered normalized counts (**B**) of specific sequences are shown for 97 genomes, presented on the x-axis in phylogenetic order according to the tree given in Figure 1. The calculations are described in the Methods section, and the underlying numbers are given in Supplemental Table S2. Calculating expectations from first-order or second-order Markov analyses (thereby taking into account dinucleotide- and trinucleotide-frequencies) produces qualitatively similar graphs (see Supplemental Figure S1 and Supplemental Table S2). Certain genomes of interest are marked with letters **a** through **w**, and their identities are given in Table 2.

To lay the foundation for a detailed assessment of exceptions to the general rule of highly overrepresented HIP1 sequences in cyanobacteria, I examined the oligonucleotide content of 97 genomes. The abundance of oligonucleotide sequences may be expressed as the count of the sequence (normalized to some standard length) or the observed number of instances in a genome divided by the number expected based on genomic characteristics (the O/E ratio). The former quantity may conceivably be closer to what is physiologically important, and the latter quantity distinguishes meaningful occurrences from those that might arise by chance and therefore indicates either selection or heightened production. The expected number of occurrences was calculated on the basis of nucleotide frequency. The O/E ratio is shown for HIP1 sequences in cyanobacteria in Figure 2A and the count of HIP1 sequences normalized to genome length shown in Figure 2B (red lines in both cases).

The broad distribution of HIP1 sites in cyanobacteria outside the picocyanobacteria (group C1) has been previously noted [4]. Equally noteworthy are the minority genomes that have relatively low HIP1 O/E ratios, despite the high ratios in related genomes. These genomes have been marked in Figure 2 and listed in Table 2. The list includes *Calothrix* PCC 7103, the strain closest to Robinson *et al.*’s HIP1-deficient *Calothrix* D253.

**Table 2:** Anomalous HIP sequences in certain cyanobacterial genomes. The calculation of normalized counts of 8-mers (Count/M) and the ratio of observed to expected counts (O/E) are described in the Methods section.

	Organism	HIP1		Top 8-mer (If Not HIP1)			Comment
		Count/M	O/E	Sequence	Count/M	O/E	
	Most cyanobacteria outside of Group C1	300–2700	55–228	-	-	-	High frequency HIP1
**a**	A: *Oscillatoria* PCC 7112	630	60	-	-	-	+HIP1 derivative (TCGATCGA)
**b**	B2: *Dactylococcopsis salina* PCC 8305	425	56	CGATCGCG	496	66	+HIP1 derivative (TCGATCGA)
**c**	B2: *Microcystis aeruginosa* NIES 843	312	42	-	-	-	Imprecise HIP1
**d**	B2: *Cyanothece* PCC 7822	111	19	-	-	-	Alternative (GCsGC)
**e**	B2: *E.turgida* EtSB endosymbiont	37	15	-	-	-	(Symbiont) Weak imprecise HIP1
**f**	B2: UCYN-A	26	15	-	-	-	(Symbiont) Weak HIP1
**g**	B2: *Geminocystis herdmanii* PCC 6308	121	44	TCGATCGA	705	70	HIP1 derivative
**h**	A: *Oscillatoria* PCC 10802	103	5	CACCGGCA	647	32	Alternative rCCGGy (DmtD)
**i**	B1: *Calothrix* PCC 7103	14	3	CAGGCGCC	159	53	Alternative (GGCGCC)
**j**	B1: *Richellia intracellularis* HH01	9	4	GCAGCAGC	30	12	(Symbiont) No high frequency 8-mer
**k**	B1: *Nostoc azollae* 0708	204	43	-	-	-	(Symbiont) Imprecise HIP1
**l**	B4: *Chamaesiphon minutus* PCC 6605	448	43	TCGATCGA	750	51	HIP1 derivative
**m**	C1-p: low-GC *Prochlorococcus* (9)	1–6	0.5–1				
**n**	C1-s: high-GC *Prochlorococcus/Synechococcus* (15)	8–171	2–5	various	52–1278	8–21	Weak oligomer (TGATCA)
**o**	C2: *Prochlorothrix hollandica* PCC 9006	711	33	CGATCGCC	753	36	Weak imprecise HIP1
**p**	*Cyanothece* PCC 7425	617	38	-	-	-	Weak imprecise HIP1
**q**	*Acaryochloris marina* MBIC 11017	285	24	-	-	-	Weak HIP1
**r**	*Leptolyngbya* PCC 6406	925	42	-	-	-	Weak HIP1
**s**	*Leptolyngbya* heron island J	475	37	-	-	-	Weak HIP1
**t**	*Synchococcus* OS Type A	23	1	GGGATCCC	1160	39	HIP1 derivative
**u**	*Synechococcus* OS Type B	19	1	GGGATCCC	1345	50	HIP1 derivative
**v**	(root): *Gloeobacter violaceus* PCC 7421	68	2	TCAAAAAA	43	15	No high frequency 8-mer
**w**	*Gloeobacter kilaueensis* JS1	102	3	TCAAAAAA	48	14	No high frequency 8-mer

### 2.3. Frequencies of Other Oligomers

**Figure 3:**
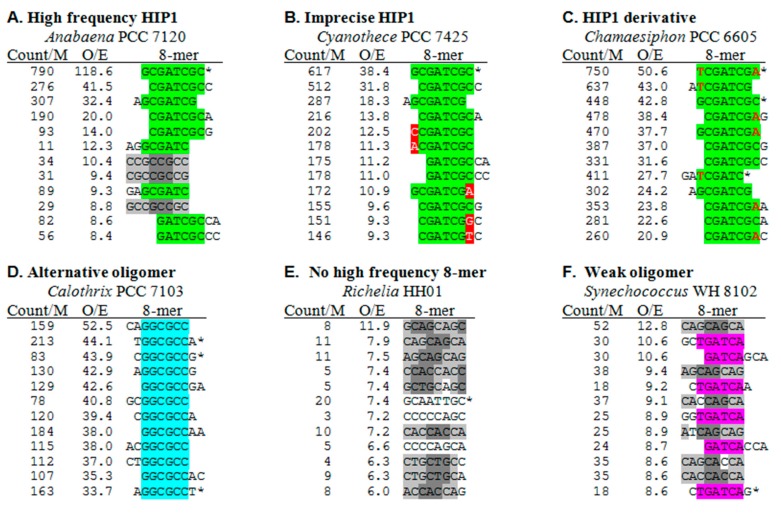
Most overrepresented 8-mers in selected genomes. Each panel shows the 12 most overrepresented 8-mers in genomes chosen to illustrate different classes. The calculations of the frequencies of the given 8-mer per million nucleotides (count/M) and the ratio of observed counts and expected counts (O/E) are described in the Methods section. Complete and partial HIP1 sequences are highlighted in green, and an overrepresented derivative of HIP1, TCGATCGA, is shown with differences from HIP1 in red font. Other, more sporadic differences from HIP1 are highlighted in red. GGCGCC sequences is highlighted in cyan and TGATCA in pink. 8-mers composed of a triplet repeat are represented in gray, with different shadings used to make the triplet repeat more clear. Palindromic sequences are marked with an asterisk. Nonpalindromic sequences represent themselves and their complement (e.g., CGATCGCC/GGCGATCG), and their frequencies are an average of the two.

In order to assess whether the cyanobacterial genomes with low HIP1 frequencies exhibit a different high frequency 8-mer, I examined the 8-mer frequencies of all the genomes. The patterns of results fall into different classes, representative samples of which are shown in Figure 3. The list of top 8-mers in *Anabaena* PCC 7120 (Figure 3A) is typical for those genomes with highly overrepresented HIP1 sequences. After the HIP1 sequence itself, the next most overrepresented 8-mers are those that overlap HIP1. At low O/E ratios, 8-mers appear that are triplet repeats. At least in the case of *Anabaena*, they occur almost exclusively in coding regions and associated with a specific reading frame, and may therefore be determined by amino acid and codon preferences.

Panels B and C of Figure 3 provide examples of the pattern of top 8-mers in genomes with lower HIP1 O/E ratios (but not necessarily low HIP1 frequencies). The top 8-mers in *Cyanothece* PCC 7425 are a mixture of overlaps (as with *Anabaena*) and single nucleotide substitutions with respect to the HIP1 sequence. In contrast, the list of *Chamaesiphon* PCC 6605 consists of two types of sequences: HIP1 and its overlaps and a palindromic relative of HIP1 (TCGATCGA) and its own overlaps.

*Calothrix* PCC 7103 (Figure 3D) is a member of a small class of cyanobacteria with genomes lacking highly overrepresented HIP1 sequences and displaying instead a different highly overrepresented sequence, in this case the 6-nucleotide sequence, GGCGCC. It is not until the 25th 8-mer (O/E = 13.9) that a sequence unrelated to GGCGCC appears in the list (not shown).

The last classes of genomes are those with no highly overrepresented sequence. The top 8-mers of *Richelia intercellularis* HH01 (Figure 3E) are mostly overrepresented triplet repeats of a sort found in all cyanobacterial genomes. *Synechococcus* WH 8102 (Figure 3F) exhibits a modestly overrepresented 6-mer, TGATCA, embedded in the top 8-mers.

The appearance in these lists of overrepresented sequences apart from HIP1 (as in Figure 3C,D) prompted me to search for such sequences in all cyanobacteria (Figure 2 and Table 2**)**. Two apparent derivatives of HIP1, both palindromic, are found highly overrepresented in a few cyanobacteria. GGGATCCC (differing from HIP1 at the 2nd and 7th nucleotides) is found as the most frequent (Supplemental Table S3) and most overrepresented (Figure 2) octanucleotide in the thermophilic cyanobacteria *Synechococcus* JA-3-3Ab and *Synechococcus* JA-2-3B (marked **t** and **u**, respectively, in Figure 2 and Table 2). TCGATCGA (differing from HIP1 at the 1st and 8th nucleotides) is similarly found as the dominant octanucleotide in two group B cyanobacteria, *Geminocystis herdmanii* PCC 6308 and *Chamaesiphon minutus* PCC 6605 (marked **g** and **l**), and as highly overrepresented octanucleotides in a few other cyanobacteria.

It is striking that the appearance of these non-HIP1 octanucleotides coincides with a depression in the frequency of HIP1 itself. That observation inspired me to look more closely at the other genomes in which the frequency of HIP1 is depressed. Examination of the frequency lists (excerpts shown in Figure 3), made clear that three genomes that lack highly overrepresented HIP1 sequences have other sequences that are highly overrepresented. GGCGCC is overrepresented in *Calothrix* PCC 7103 (marked **i**), as well as in the 19301 nt available for *Calothrix* D253. The degenerate sequence [ag]CCGG[ct] (rCCGGy) is overrepresented in *Oscillatoria* PCC 10802 (marked **h**) and GC[gc]GC (GCsGC) in *Cyanothece* PCC 7822 (marked **d**). These sequences are markedly overrepresented only in these three genomes (Supplemental Table S2).

**Figure 4:**
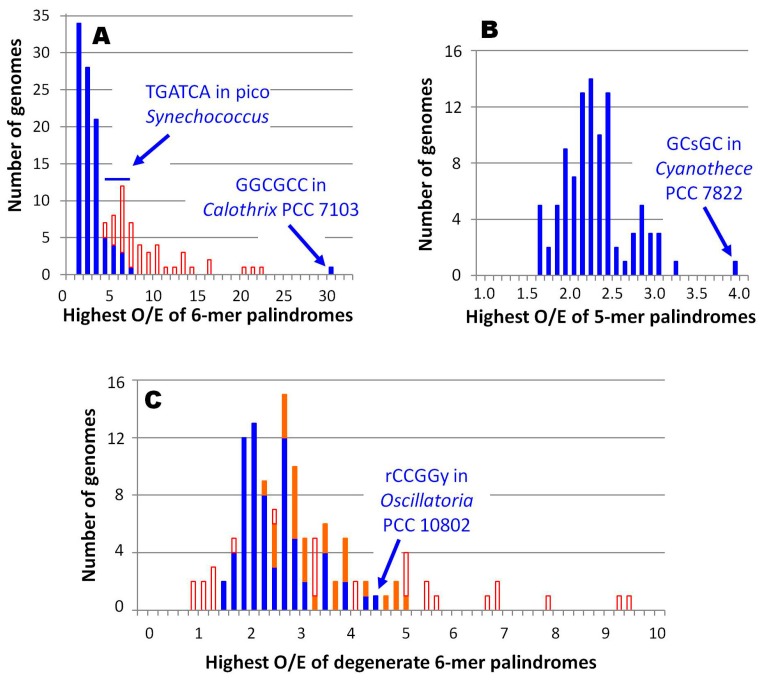
Distributions of most overrepresented oligomers in cyanobacterial genomes. The most overrepresented of a specific class of oligomer was determined for each of 97 genomes. Filled boxes: number of most overrepresented oligomers not contained within HIP1. Unfilled boxes: number of most overrepresented oligomers contained within HIP1 (visible only when the number is higher than filled boxes). (**A**) Nondegenerate palindromic 6-mers, with a bin size of 1; (**B**) Palindromic 5-mers (the central nucleotide is either [AT] or [CG]), with a bin size of 0.1; (**C**) Degenerate palindromic 6-mers, with a bin size of 0.2. Oligomers not contained with HIP1 have been split between those in Group C1 and *Gloeobacter* (red) and those in other groups (blue).

There is nothing unusual about finding an overrepresented oligomer in a genome, but these three stand out as remarkable outliers. GGCGCC in *Calothrix* PCC 7103 is by far the most overrepresented 6-mer in all 97 genomes, 4-times as overrepresented as the next palindromic hexamer apart from the central 6-mer of HIP1 (Figure 4A). The O/E ratio of GGCGCC in the 19,301 nt available for *Calothrix* D253 is slightly lower — 15.5 — but it is still a decided outlier. Similarly, GCsGC of *Cyanothece* PCC 7822 is the most overrepresented palindromic 5-mer found in any of the cyanobacterial genomes (Figure 4B). Finally, rCCGGy of *Oscillatoria* PCC 10802 is the most overrepresented degenerate 6‑mer palindrome amongst all cyanobacteria outside of the picocyanobacteria, if one excludes those 6‑mers contained within HIP1 (Figure 4C).

To summarize, of the ten cyanobacterial genomes outside of Group C1 that have the lowest frequency of HIP1 (whether judged by O/E or Counts/M), two have highly overrepresented sequences related to HIP1 and three have other highly overrepresented sequences that are nearly unique in their classes. The frequencies of these sequences are comparable to the high-frequency HIP1 sequences (Figure 2B)—around 500–2500 instances per million nucleotides (except rCCGGy, which is higher). What about the other five genomes? Two are strains of *Gloeobacter*, which lies outside the main radiation of cyanobacteria [12]. The other three, however, are solidly within Group B. Interestingly, all three are obligate symbionts: EtSB (**e**), UCYN-A (**f**), and *Richelia intercellularis* HH01 (**j**). The symbiotic *Nostoc azollae* 0708 (**k**) also has a low frequency of HIP1 sequences relative to its phylogenetic neighbors. The genome of the fifth Group B symbiont, *Prochloron didemni* P4, is exceptional in its high frequency of HIP1 sequences, as it is exceptional in many other regards [17].

The genomes of Group C1 cyanobacteria differ in many respects from those of other cyanobacteria, so perhaps it is not surprising that they do not have highly overrepresented HIP1 sequences. However, in many within this group, the hexamer with the highest O/E ratio is another sequence with GATC at its core, TGATCA (Figure 4A). The low-GC Prochlorococcus strains and the chromophore from *Paulinella* lack any highly overrepresented 8-mers.

### 2.4. Extensions to HIP1 and Other High Frequency Oligomers

The fact that octanucleotides that overlap HIP1 sequences often have comparable O/E ratios as HIP1 itself (e.g., Table 2, **b** *Dactylococcopsis salina*) suggested that the functional sequence may sometimes be longer than 8 nucleotides. Accordingly, sequences flanking instances of HIP1 were examined iteratively to determine whether any positions outside of the canonical 8 were well conserved (Table 3). In 12 genomes, HIP1 sequences showed extensions that occurred 4 to 15 times more frequently than expected by chance. In the case of *Synechococcus* PCC 6803, 88% of the HIP1 sequences were embedded in the same 10-nucleotide sequence. All of the extensions preserved the palindromic nature of HIP1 sequences.

The same procedure was applied to the non-HIP1 sequences identified in the previous section. In one genome, that of *Oscillatoria* PCC 10802, the dominant repeated sequence, rCCGGy was found to be embedded in a larger 8-nucleotide sequence, although this is the case only with two sequences (ACCGGC and GCCGGT) within the pattern.

**Table 3:** Extensions to HIP sequences. Pairs of upper case letters in the extended HIP sequence indicate that at least 50% of enclosed sequences are extended as shown. For example, $\underline{G}$GCGATCGC$\underline{C}$, indicates that C_GGCGATCGCC_/C_GCGATCGC_ is greater than 0.5, where C**_S_** is the count of sequence **S**. Pairs of lower case letters indicate that at least 25% of enclosed sequences are extended as shown and the O/E ratio of that extension is greater than 4. When two percentages are given for the Extended HIP/HIP ratio, the first number is for the inner extension and the second number is for the outer extension.

Organism	Extended HIP	Extended HIP/HIP
B2: *Cyanobacterium aponimium* PCC 10605	t$G\underline{\text{CGATCG}}C$a	43%
B2: *Cyanobacterium stanieri* PCC 7202	g$G\underline{\text{CGATCG}}C$c	34%
B2: *Synechocystis* PCC 6803	G$G\underline{\text{CGATCG}}C$C	88%
B2: *Leptolyngbya* PCC 7376	G$G\underline{\text{CGATCG}}C$C	69%
B2: *Synechococcus* PCC 7002	G$G\underline{\text{CGATCG}}C$C	71%
B2: *Dactylococcopsis salina* PCC 8305	gc$g\underline{\text{CGATCG}}c$gc	(30%, 12%)
B2: *Halothece* PCC 7418	gC$G\underline{\text{CGATCG}}C$Gc	(63%, 31%)
A: *Oscillatoria acuminate* PCC 6304	g$G\underline{\text{CGATCG}}C$c	27%
D: *Geitlerinema* PCC 7407	g$G\underline{\text{CGATCG}}C$c	36%
D: *Leptolyngbya* heron island J	t$G\underline{\text{CGATCG}}C$a	45%
E: *Acaryochloris marina* MBIC 11017	T$G\underline{\text{CGATCG}}C$A	56%
C2: *Prochlorothrix hollandica* PCC 9006	g$g\underline{\text{CGATCG}}c$c	30%
B1: *Oscillatoria* PCC 10802	c$\underline{\text{ACCGGC}}$a/t$\underline{\text{GCCGGT}}$g	33%

### 2.5. DNA Methyltransferases Associated with HIP1 and Other Oligomers

#### 2.5.1. GATC Methyltransferases and Their Target Sites

**Figure 5:**
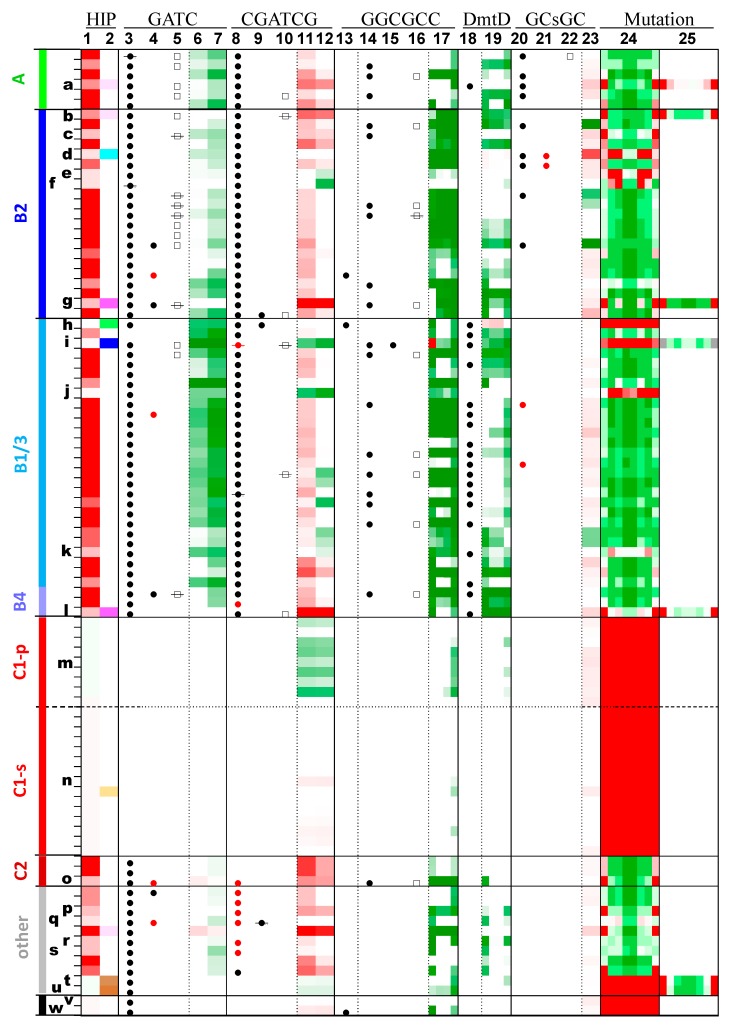
Occurrences of highly iterated palindromes and the enzymes that may recognize them.

As previously noted [4], *dmtA*, a gene capable of encoding a type II alpha m6A MTase that recognizes GATC within HIP1 sites, is found in almost all cyanobacteria outside of Group C1 (Figure 5, column 3). The sole exceptions are *Mastigocladopsis repens* PCC 10914, whose incomplete genome sequence shows no sign of any recognizable GATC-specific MTase, *Trichodesmium erythreum* IMS101, whose gene is interrupted at the 17th codon by an apparent retroelement (thereby deleting the conserved Motif X), and UCYN-A, whose gene suffers from a frame shift that leads to the loss of the final 51 amino acids. Even the two *Gloeobacter* strains possess DmtA orthologs. The phylogeny of the DmtA proteins overall does not match the phylogeny of the strains (Supplemental Figure S2A), indicating multiple instances of horizontal gene transfer. Some genomes have two versions of GATC-methylating proteins (Figure 5, columns 3 and 4), in some cases similar to the highly unusual enzyme DmtE [7].

In Figure 5, each row offers for a single genome a graphical representation of the frequencies of certain oligomers (columns with underlined labels) and the presence or absence of MTases and Res (other columns). The genomes are listed in phylogenetic order, the same order as in Figure 1 and Figure 2, with the same group names. Certain genomes are marked with letters as described in Figure 2 and Table 1. The color schemes are described in more detail in the Methods section, but in brief, measures of oligomer frequency are shown on a scale from bright red (overrepresentation) to dark green (underrepresentation). The presence of MTases and REases are indicated with circles (filled for MTases, empty for REases). If a circle has a line through it, then the gene of the corresponding enzyme has a frame shift or deletion that most likely renders the enzyme nonfunctional. Red symbols indicate atypical MTases, DmtE-like MTases (GATC column) or m6C-MTases (other columns). The columns are as follows:

- HIP: (1) O/E ratio of HIP1, (2) O/E ratio of a second highly iterated palindrome, as indicated in Table 2 and Figure 2.
- GATC: (3) GATC-specific MTase, (4) Second GATC-specific MTase, (5) GATC-specific REase, (6) O/E ratio of GATC, (7) O/E ratio of GATC (HIP1 subtracted).
- CGATCG: (8) CGATCG-specific MTase, (9) Second CGATCG-specific MTase, (10) CGATCG-specific REase, (11) O/E ratio of CGATCG, (12) O/E ratio of CGATCG (HIP1 subtracted).
- GGCGCC: (13) GGCGCC-specific MTase, (14) GrCGyC-specific MTase, (15) Second GrCGyC-specific MTase, (16) GrCGyC-specific REase, (17) O/E ratio of sequences specified by GrCGyC: GGCGCC, GGCGTC, GACGCC, GACGTC.
- DmtD: (18) rCCGGy-specific MTase, (19) O/E ratio of sequences specified by rCCGGy: GCCGGC, GCCGGT, ACCGGC, ACCGGT.
- GCsGC: (20) GCsGC-specific MTase, (21) Second GCsGC-specific MTase, (22) GCsGC-specific REase, (23) O/E ratio of GCsGC.
- Mutation: (24) Fraction of sequences deviating from HIP1 at positions 1 through 8, (25) Fraction of sequences deviating from a second highly iterated palindrome (positions 1 through 8), as indicated in Table 2 and Figure 2. For example, the second position is calculated as (**C_GnGATCGC_**−**C_GCGATCGC_**)/**C_GnGATCGC_**, where **C_S_** is the counts of the sequence or sequence pattern **S**, and **n** represents any nucleotide.

Ten genomes also possess genes that are predicted to encode restriction enzymes that cut GATC (Figure 5, column 5). An additional six genomes have similar genes that are clearly nonfunctional. The restriction enzymes (REases) are found within Groups A and B, and are associated with two branches of the GATC MTase tree (Supplemental Figure S2A).

GATC sequences are generally underrepresented in Group A and B cyanobacteria (Figure 5, columns 6 and 7 and Supplemental Table S4), though there is a great range in the degree of underrepresentation. There is no obvious general relationship between the frequency of GATC sites and the frequency of HIP1 sequences, the number of GATC-specific MTases, or the presence of a GATC-specific REase. However, it may be worth noting that the most extreme case (by a good margin) is *Calothrix* PCC 7103 (marked **i**), which has only 16% of the expected number of GATC sites and also possesses a gene that potentially encodes an active GATC restriction enzyme. That strain also has the lowest frequency of HIP1 in the Group A and B cyanobacteria.

#### 2.5.2. CGATCG Methyltransferases and Their Target Sites

MTases capable of methylating CGATCG appear to be almost coextensive with the presence of overrepresented HIP1 sites (Figure 5, column 8). There are six exceptions: four cyanobacteria outside of Groups A, B, and C apparently lacking a gene recognizable as encoding a CGATCG MTase, and two Group B1 cyanobacteria with mutations in such a gene. First, the gene from *Scytonema hofmannii* has a frame shift (or sequencing error) at the 5' end of its gene. Second, the gene from *Calothrix* PCC 7103 (**j**) has evidently suffered a deletion event that eliminates the last 40% of the gene as well as the first 40% of an adjacent gene whose hypothetical product is similar to CGATCG-specific endonucleases. Most of the MTases are orthologs of DmtC of *Anabaena* PCC 7120 [7], a m5C MTase (methylates the 5-carbon of cytosine). However, there are several instances of m4C CGATCG-MTases (methylating the 4-nitrogen of cytosine and quite distinct structurally from m5C cytosine MTases [20]), mostly outside Groups A and B.

Outside of Groups A and B, those strains that lack high frequency HIP1 sites also lack CGATCG MTases. There are also four strains outside of A and B that have high frequency HIP1 sites but nonetheless do not have a recognizable CGATCG MTase: *Synechococcus elongatus* PCC 6301 and PCC 7942 (both Group C1), *Pseudanabaena* PCC 7367, and *Geitlerinema* PCC 7407. There is nothing obvious that sets these organisms apart except that the first three have the highest absolute densities of CGATCG (not O/E ratio) amongst all cyanobacteria considered, and *Geitlerinema* is not far behind (Supplemental Table S3). Cyanobacteria in Groups A and B generally have m5C CGATCG MTases, while those in other groups have m4C CGATCG MTases.

CGATCG sequences are generally overrepresented in strains carrying overrepresented HIP1 sequences (Figure 5, column 11). Perhaps this is not surprising, as it is the expected result from gross overrepresentation of the HIP1 sequences that contain them. Indeed, when HIP1 sequences are subtracted out, the overrepresentation largely disappears (Figure 5, column 12). Two exceptional cases stand out: *Calothrix* PCC 7103 and *Richelia* HH01, both of whom lack overrepresentation of HIP1, have highly depressed levels of CGATCG.

#### 2.5.3. Other Methyltransferases and Their Target Sites

If cyanobacterial genomes that possess high frequency HIP1 sites almost always carry two MTases that recognize the sequence (one recognizing GATC, the other CGATCG), what about those genomes that have different high frequency sites besides HIP1? The genome of *Calothrix* PCC 7103 (**i**) is in this category, exhibiting a high frequency of the sequence GGCGCC. Figure 5, column 13, shows that it does not have a recognizable GGCGCC-specific MTase, and none of the cyanobacteria considered in this study carry a recognizable GCGC-specific MTase. However, the *Calothrix* strain is among many cyanobacteria that possess a GrCGyC-specific MTase (column 14), whose specificity includes GGCGCC; indeed, it is the only cyanobacterial strain to have two such MTases (column 15). As is typical, the phylogenetic tree of these MTases does not accord with the organismal tree, and the two *Calothrix* PCC 7103 MTases lie on different branches of the tree. The presence of such a MTase (with or without the corresponding REase) is correlated with a marked deficiency in sites recognized by the enzyme—true in 78% of the cases (column 17). The others have moderate deficiencies, except for one—*Calothrix* PCC 7103. That strain is unique in that one of the recognition sites, GGCGCC, is highly overrepresented. The other three sites are either moderately (GACGCC, GGCGTC) or strongly (GACGTC) underrepresented. The *Calothrix* strain, like most Group A and B cyanobacteria, therefore has a MTase that modifies a short DNA sequence that is unusually abundant, however the specificity of the MTase is less strict than necessary.

Most Group B and some Group A cyanobacteria possess an ortholog of the m5C MTase, DmtD [7], specific for rCCGGy (Figure 5, column 18), a highly overrepresented sequence of *Oscillatoria* PCC 10802 (marked **h**). In most genomes of strains carrying a DmtD ortholog, all four sequences encompassed by rCCGGy are underrepresented, sometimes very much so (column 19 and Supplemental Table S4). The *Oscillatoria* strain is exceptional in that three of the four specifications of rCCGGy (GCCGGC, GCCGGT, and ACCGGC) are highly overrepresented.

*Cyanothece* PCC 7822 (marked **d**) has a low O/E ratio for HIP1 but a remarkably high ratio for the sequence GCsGC. It carries a predicted MTase with GCsGC specificity (Figure 5, column 20). The GCsGC sequence is mildly overrepresented in most cyanobacteria, regardless of the presence of the MTase, but none come close to the degree of overrepresentation in *Cyanothece* PCC 7822 (column 23 and Supplemental Table S4). This strain and its sister *Cyanothece* PCC 7824 both have two phylogenetically distinct versions of a GCsGC-specific MTases (columns 20 and 21), but the level of GCsGC in the latter strain is within the normal range.

In short, all cyanobacterial genomes with highly overrepresented sequences have at least one putative MTase capable of methylating those sequences.

### 2.6. Substitution Patterns

An analysis of deviations from highly iterated sequences has proven helpful in determining how these sequences arise and are maintained [6,21]. As previously shown (Figure 3), one-off sequences are common in many cyanobacteria. Figure 5, column 24, shows that there is a marked preference in most genomes against deviation at the 4th and 5th positions (GCGATCGC). The only exceptions are those genomes with the lowest HIP1 O/E ratios, *i.e.*, those outside the phylogenetic clades with overrepresented HIP1 (**m**, **n**, **v**, and **w**), a symbiont (*Richelia intracellularis*, **j**), and four cyanobacteria with different highly overrepresented oligomers: *Oscillatoria* PCC 10802 (**h**), *Calothrix* PCC 7103 (**i**), and Synechococcus OS Types A and B (**t** and **u**). The latter two are of particular interest, since there is a strong bias against deviation at the same positions within the genome's overrepresented GGGATCCC (Figure 5, column 23), indicating that there may be selection against deviation at the methylation position of GATC but only when the sequence is part of HIP1 or another overrepresented oligomer. This idea gains further support by the finding that there is no similar bias against deviation from the central AT within TGATCA nor within CGATCG when the 6-mer is not flanked by G on the left and C on the right (Supplemental Table S4).

There is also a strong tendency for deviation from HIP1 to be greatest at the 1st and 8th positions, associated, reasonably enough, with those genomes in which CGATCG is most overrepresented (column 11). Part of this phenomenon may be trivially explained by the presence of two dominant 8-mers (GCGATCGC and TCGATCGA), differing at the 1st and 8th positions. However, this explanation accounts for only a small fraction of cases.

## 3. Discussion

### 3.1. The Nature of HIP Sequences

The nature of highly iterated palindromic sequences in cyanobacteria appears to be considerably more complex than one might have expected. HIP1 sequences are overrepresented in most cyanobacteria outside of Group C1, but not in all The dominant 8-mer is usually GCGATCGC but the sequence may be extended in both directions, and there are derivatives of HIP1 that have replaced the canonical sequence. In rare cyanobacteria (one of them chanced upon by Robinson *et al.* [3]), there are other quite different highly iterated palindromes whose degrees of overrepresentation are as unusual as those of HIP1. Perhaps we should refer to HIP sequences as a class of overrepresented oligomers of which HIP1 is the prime example.

HIP sequences that differ from HIP1 share certain characteristics. They are seen only in genomes where HIP1 is low or nearly absent. Like chi sites [1], they are GC-rich, either 100% GC (GGCGCC and GCsGC) or at least 75% GC (GGGATCCC and rCCGGy). The exceptional sequence TCGATCGA (50% GC) supplements HIP1 in some cyanobacteria but was never observed to replace it. They generally occur in the range of 1000 to 2000 instances per million nucleotides, similar to the range of HIP1 (Figure 2B).

### 3.2. The Nature of the Proteins Identified as Methyltransferases

One of the key findings of this study is that every genome with HIP sequences also possesses one or more MTases predicted to recognize some portion of the sequence. In judging how much weight to place on this observation, it is important to acknowledge that the study considers genomes, not enzymes, and predicted MTases, seldom proven MTases. However, there is good reason to believe that the activities ascribed here to the MTases are accurate. First, all of the putative GATC-specific m6A type α MTase activities were predicted by REBASE, through an algorithm that considers both overall similarity to proven enzymes and similarity of the target recognition domain (see Methods). These MTases, are orthologs or paralogs of the proven GATC-specific MTase DmtA of *Anabaena* PCC 7120 [7] and of *Synechocystis* PCC 6803 [22]. Genomic DNA isolated from seven Group B cyanobacteria and one Group C2 cyanobacterium are resistant to cleavage by the GATC-specific restriction enzyme MboI [23,24]. The MTases with experimental evidence are found in each of the four major groupings of phylogenetic groups (Supplemental Figure S2A).

MTases identified as CGATCG-specific have gained less attention from the research community, but the little available *in vivo* evidence is consistent with the assignments in Figure 5: The CGATCG-specific restriction enzyme PvuI fails to cut genomic DNA isolated from three Group B cyanobacteria but does cut genomic DNA from Group C2 cyanobacterium *Synechococcus* PCC 7942, as expected [3,8,24]. The evidence from the sequences is more compelling. The m5C CGATCG MTases are orthologs of the proven CGATC-specific MTase DmtC of *Anabeana* [7] and of *Synechocystis* PCC 6803 [8] and form a coherent group that includes CGATCG-specific MTase XorII from the heterotrophic *Xanthomonas oryzae* but excludes other m5C MTases, such as DmtD (rCCGGy) and AvaII (GGwCC) from *Anabaena* PCC 7120 (Supplemental Figure S2B). It is therefore likely that all the MTases in the group recognize CGATCG.

Finally, the conservation of both MTases over a broad range of cyanobacteria indicates strongly conserved functions.

### 3.3. Functional Roles of Methyltransferases Associated with HIP Sequences

The starting point for this study was the observation that HIP1 sequences in cyanobacteria are almost coextensive with the presence of GATC-MTases [4]. That by itself is not completely satisfying, as there are many unrelated phenomena that track the phylogeny of cyanobacteria. It is the exceptional cases that offer the hope of insight. Here are the three exceptional cases presented in this study: *Calothrix* PCC 7103 lacks the highly overrepresented HIP1 sequences typical of its kindred cyanobacteria and instead has highly overrepresented GGCGCC sequences. At the same time, it has recently lost its CGATCG-specific MTase and gained two MTases capable of methylating GGCGCC. *Cyanothece* PCC 7122 is also near the bottom of the list regarding HIP1 overrepresentation but at the very top of the list in overrepresentation of palindromic 5-mers with GCsGC. Although the strain has retained its CGATCG-specific MTase, it has gained two GCsGC-specific MTases. *Oscillatoria* PCC 10802 is the third telling of the same story: low HIP1, high rCCGGy, gain of rCCGGy-specific MTase. For each strain, there is the same improbable constellation of events, and it is beyond belief that they should occur together three times by chance. This strikes me as the best of the circumstantial evidence we have that there is a strong functional relationship between HIP sequences and their corresponding MTases.

What that relationship may be remains a mystery. One place to begin is to frame the problem through consideration of the little biochemical evidence at hand. First, GATC-methylation (in *Anabaena* PCC 7120) [7] and CGATCG-methylation (in *Synechocystis* PCC 6803) [8] appear to be required for viability under certain laboratory conditions. If methylated HIP sequences perform a single selectable function, then that function cannot confer merely a long-term advantage. For example, functioning as a DNA uptake recognition signal would not be required for viability in the short term. A role for methylated HIP1 sites in homologous recombination, analogous to that played by chi sites [1], is consistent with the short-term requirement for the two MTases, since recombination enzymes RecA [25] and LexA [26] are also known to be required for viability in at least some cyanobacteria under some laboratory conditions. The essentiality of recombination, not seen in many bacteria, is not surprising in cyanobacteria, organisms that live by photosynthesis, a process that generates DNA-damaging oxygen radicals [27]. Analogies aside, there is no evidence linking HIP1 to homologous recombination.

In contrast, there ***is*** evidence linking HIP1 sequences (or internal sites) to site-specific recombination [5,6]. Furthermore, the directed cutting at HIP1 sites could partially explain the precise insertion of the repeat module SDR5 between the third and fourth nucleotides of HIP1 [28]. However, it is not clear how site-specific recombination could relate to the short-term requirement for GATC- and CGATCG-methylation. One must seriously entertain the idea that the actors are playing multiple roles.

Then there is the phylogenetic evidence that HIP1 sites have been rapidly lost and wholly different HIP sites rapidly gained, as judged by the three exceptional genomes and those of closely related cyanobacteria. It is too much to expect that a hypothetical protein that recognizes HIP1 could so easily change its binding specificity. An alternative view is that the function of HIP1 is useful but not essential, that the MTases are playing multiple roles, some essential and some not, and that the function of the new HIP sequences in the exceptional strains have no function at all. This idea is explored in the next section.

### 3.4. How are HIP Sequences Gained?

There are few MTase roles better understood than that played in methylation-directed mismatch repair (MMR) of DNA [29], in which methylation of the template strand at a replication fork biases repair of mismatches towards the original DNA sequence. In *E. coli*, MMR requires the action of three proteins MutS, MutL, and MutH [30,31]. The latter nicks mismatched DNA near hemimethylated G^me^ATC/GATC, required for repair. While MutS and MutL are found universally, including in cyanobacteria, MutH is confined to a group of gamma-proteobacteria that includes *E. coli*. It is not completely understood how bacteria outside this group identify the methylated DNA required.

I propose that in cyanobacteria outside of Group C1, MMR is directed by G^me^C, where the methylated cytosine is usually contributed by a CGATCG-MTase that methylates the first cytosine. This sequence does not contain GC, so methylation is effective only if the recognition sequence is preceded by G or followed by C. In *E. coli*, the efficiency of MMR begins to fall off when the methylation site is separated from a mismatch by about 1000 nt [30,32]. If the same is true in cyanobacteria, and there is selection for effective MMR, then G^me^CGATCG or CGATCG^me^C sequences (the two 7-nt sequences within HIP1) should accumulate in their genomes up to a density of about once every 1000 nucleotides. The observed frequencies of HIP1 sites and alternate HIP sites in cyanobacteria (Figure 2A) are very close to this value (except for a higher value for *Oscillatoria* PCC 10802), and the frequencies of the 7-mer sequences contained within HIP1 are almost the same.

**Figure 6:**
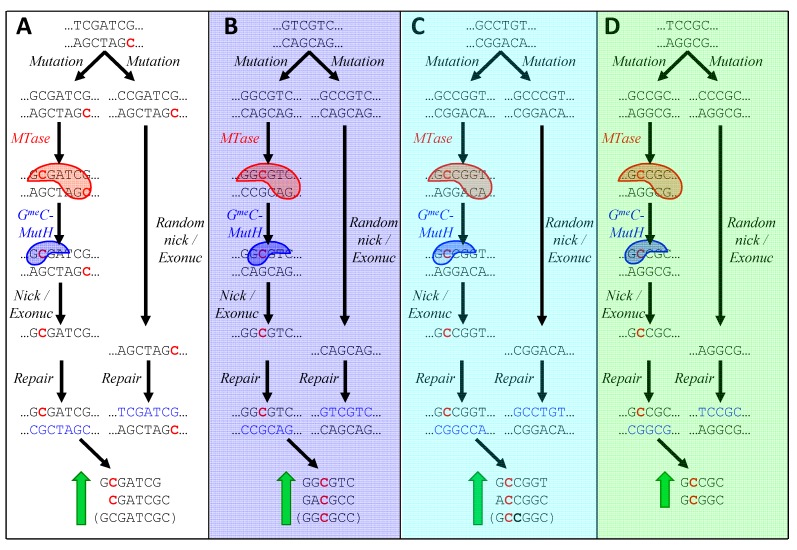
Model for the biased creation of new HIP sequences. In each panel, a sequence containing a MTase recognition site (or a near miss) is presumed to have just been replicated. The lower strand is the parental strand and the upper strand is newly synthesized. Pre-existing recognition sites are therefore hemimethylated, with one methylated cytosine (red). Two mutations are shown as taking place during replication. In the left-hand case, the mutation leads to a G lying next to the C at the MTase's methylation site. The C is acted on by the MTase (red blob. Strands with $G^{\text{me}}C$ are then recognized by a hypothetical G^me^C-specific nicking enzyme (blue blob; analogous to MutH in *E. coli*), which nicks the strand opposite the methylation. This allows MutL/MutS to degrade the opposite strand and DNA polymerase to synthesize a new strand using the mutated upper strand as the template. The sequences at the bottom of each panel are those produced by the described mechanism. Sequences in parentheses are special cases (see text). (**A)** Mutation of nucleotide to the left of CGATCG in a typical cyanobacterium; (**B**) Mutation of a sequence one off from GrCGyC in *Calothrix* PCC 7103 (**i**); (**C**) Mutation of a sequence one off from rCCGGy in *Oscillatoria* PCC 10802 (**h**); (**D**) Mutation of a sequence one off from GCsGC in *Cyanothece* PCC 7822 (**d**). Letters in parentheses refer to symbols in Table 2.

Figure 6A illustrates the process by which G^me^C-dependent MMR might lead to an increase in sequences one off from HIP1. A CGATCG site in a typical cyanobacterium has just been replicated, yielding a parental, methylated strand and a new, unmethylated strand. The panel depicts two cases in which a mutation has occurred during replication just to the left of the CGATCG site. In the left-hand case, the nucleotide has mutated to a G, producing a G^me^C site recognized by the postulated G^me^C MutH analog. The binding of this protein directs MMR to nick and degrade the opposite strand, thereby preserving the mutation. In contrast, a mutation to any other nucleotide does not produce a G^me^C site, and the mismatch is resolved at random (or the mutation is preferentially lost if it happens to lie within ~1000 nt of a distant G^me^CGATCG site). As a result, the number of G^me^CGATCG sites increases.

The existence of G^me^C-dependent MMR would also explain how the alternative HIP sequences might arise. In the case of *Calothrix* PCC 7103 (**i**; Figure 6B), a site close to that recognized by the strain’s resident GrCGyC MTase is mutated in two hypothetical manners: the left-hand case producing GGCGyC and in the right-hand case producing GCCGyC. Only in the first case does MMR favor the mutation, leading to the accumulation of those instances of GrCGyC that contain G in place of r (or, equivalently, C in place of y).

Figure 6C shows how only certain instances of rCCGGy in *Oscillatoria* PCC 10802 (**h**) could accumulate as a result of the action of G^me^C-directed MMR and a rCCGGy-specific MTase. Similarly, in Figure 6D, mutations in *Cyanothece* PCC 7822 (**d**) to GCsGC are selected in the presence of a GCsGC-specific MTase.

**Figure 7:**
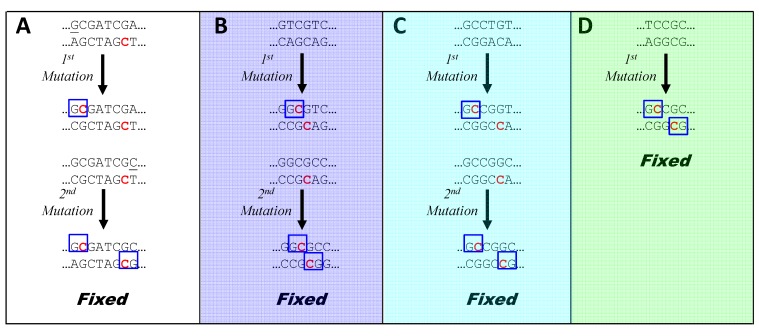
Model for the fixation of new HIP sequences. A sequence is termed “fixed” when both strands are subject to protection from further mutation by MMR. In each panel, a sequence containing a MTase recognition site (or a near miss) undergoes biased mutation through the mechanism shown in Figure 6, where the upper and lower strands are daughter and parent, respectively. If both strands of the resulting sequence do not contain a G^me^C site (blue box), then the resulting sequence undergoes a second round of mutation by the same mechanism, at the end of which both strands have G^me^C sites. Methylated sites are shown in red. Panels are as in Figure 6.

This model predicts that mutation near CGATCG sites, biased by G^me^C-dependent MMR, functions as a unidirectional ratchet, leading to sequences one-off from HIP1. A second round of mutation, using the same mechanism (Figure 7A) leads directly to HIP1. This accounts for the observation (Figure 5, column 24) that sequences that are one-off from HIP1 deviate most at the first or last position. The model accounts for why CGATCG sites in most genomes aren’t particularly overrepresented except within the HIP1 context (Figure 5, columns 11 and 12). The ratchet biases mutations towards HIP1 until saturation is reached at about one site every 1000 nucleotides. At that point, most nucleotides in the genome are within 1000 nucleotides of G^me^C and an additional site would not alter the course of MMR.

The model also explains how the three exceptional HIP sequences may arise. One round of mutation biased by G^me^C-dependent MMR produces directly one of the exceptional sequences, GCsGC (Figure 7D). In the other two cases (Figure 7B and Figure 7C), it produces three of the four instances of the degenerate 6-mer: (GGCGTC, GACGCC, and GGCGCC) and (GCCGGT, ACCGGC, and GCCGGC), A second round of biased mutation leads to just the palindromic instances: GGCGCC and GCCGGC. These sequences can also be reached in a single round of biased mutation (e.g., GGTGCC to GG^me^CGCC).

A major inference from this model is that the gross overrepresentation of the exceptional sequences does not imply selection for their function. The overrepresentation may be an epiphenomenon, an incidental byproduct of the MMR machinery. While the abundance of G^me^C sites associated with the exceptional sequences may serve the organism as the basis of conventional MMR, the specifics of the sequences may have no other importance. Likewise, it is possible that HIP1 sequences have no biological importance, arising merely as a result of G^me^C-dependent MMR acting on CGATCG. The evidence concerning site-specific recombination might equally well be interpreted as based on CGATCG as on GCGATCGC. However, there are two compelling arguments for the idea that HIP1 sequences themselves have biological function. First, there are some genomes with overrepresented HIP1 sequences but no CGATCG MTase (Figure 5, columns 1 and 8), hence no postulated mechanism to account for the overrepresentation. Second, exceptional HIP sequences occur sporadically, not in clades that one would expect if the loss of HIP1 were not accompanied by a loss of function. Presumably these genomic anomalies have arisen many times over evolutionary times, but only HIP1—not the exceptional HIPs—persist. The simplest explanation is a selection for HIP1.

Potential dissonances between the model and reality should be noted. First, the predicted fixation at the palindrome is observed in the case of *Calothrix* PCC 7103 (Figure 5, column 17), but the pattern of rCCGGy in *Oscillatoria* PCC 10802 is consistent with only one round of mutation (Figure 5, column 19). Why should two rounds be favored in one instance and only one in the other?

A second potential problem with the model are the sites of methylation. The model requires that CGATCG be methylated at the first C, but the methylation site has not been determined for any MTase with CGATCG specificity [33]. There is weak experimental evidence in favor of the first C in the *Synechocystis* PCC 6803 enzyme [8], and it would not be difficult to resolve the matter by sensitivity of specific sites within genomic DNA to Sau3AI (recognizing GATC and blocked by C-methylation). The model also requires that the GrCGyC MTase of *Calothrix* PCC 7103 be methylated at the internal C, but the methylation site is known for no enzyme with this specificity [33]. The methylation site of the rCCGGy MTase ***is*** known in one case, *Anabaena* PCC 7120 [7], and it is indeed the first C as required by the model. No doubt it is the same for the highly similar enzyme of *Oscillatoria* PCC 10802. The methylation site of the GCsGC MTase of *Cyanothece* PCC 7822 is irrelevant, as either possible site fits with the model.

The model also requires in some cases that a MTase act on heteroduplex DNA that contains a recognition sequence on one strand but not the other (e.g., Figure 6, panel D). Only a few m5C MTases have been examined with regard to their abilities to methylate heteroduplexes [34,35,36]. In these instances methylation of C is efficient opposite a mismatched A. Much more is known about the abilities of REases to cut mismatched target sequences [37]. Many REases are able to cut heteroduplexes though the position of the mismatch is often important. It would therefore not be surprising if the MTases pertinent to the model were able to methylate DNA with a recognition sequence on only one of the two strands, as required.

Another issue is that the model calls for MMR to act in a manner different from what has been previously described. MMR is viewed as a mechanism to diminish the rate of mutation, not selectively increase it. More seriously, it relies on prior methylation of the parental strand rather than new methylation of the daughter strand. Inherent in the model is a race between methylation by the MTase on one hand and on the other, random resolution of the mismatch by MutS/MutL in the absence of methylation. In *E. coli* at least, the MTase generally loses.

Finally, it is necessary to explain why the phenomenon predicted to occur in the three exceptional strains does not also occur in many other cyanobacterial with the same MTases. These issues are addressed in the next section.

### 3.5. How is HIP1 Lost? How Are New HIP Sequences Selectively Gained?

The model described in the previous section relies on bias introduced by MMR to increase specific sequences up to a density (~1 site per 1000 nt) where MMR acts equally on all nucleotides of the genome. What happens when the density drops below that level? The density evidently did drop in *Calothrix* PCC 7103 (see the low level of both HIP1 and CGATCG sites in Figure 5, columns 1, 11 and 12, **i**). This was likely the result of an infection by a GATC-specific REase (column 5), drastically driving down the frequency of GATC sites (columns 6 and 7) [38,39] and an infection by a CGATCG-specific REase (column 10), now non-functional. The latter REase evidently brought with it an unusual m4C CGATCG-specific MTase, which replaced the standard m5C version (column 8). It isn’t clear whether such a methylase could functionally replace the m5C MTase in G^me^C-dependent MMR. Perhaps in most cases, the level of HIP1 sites would recover to the set point (perhaps by the mechanism proposed in Figure 6 and Figure 7) following the loss of the REases, which appear [40,41] and disappear (Figure 5, columns 5, 10 and 16) rapidly in evolutionary time.

In this case, however, during the window of opportunity, *Calothrix* gained a second copy of an MTase recognizing GrCGyC. This last event may have been critical. The overexpression of the GATC-MTase underlying MMR in *E. coli* can increase methylation at the replication fork [29]. In *E. coli*, this leads to methylation signals on both strands and the loss of biased MMR. In *Calothrix*, however, the parental strand does not carry the postulated G^me^C signal (see Figure 6), so an increase of expression of the GrCGyC MTase could ***enable*** biased MMR for the cases shown in Figure 6 and Figure 7 (while reducing the efficiency of conventional MMR). Since according to the model, it doesn’t matter where the G^me^C required for MMR comes from, the ratchet might act on the temporarily more prevalent GrCGyC sites until GG^me^CGCC reaches a density where all nucleotides are near G^me^C sites and bias no longer operates to produce new G^me^C sites. In this process, the expression of the GrCGyC MTase must be high enough to lead to some methylation of the nascent strand at a replication fork but not so high as to reduce too much the efficiency of conventional MMR. At the outset, with few G^me^C sites, the calculus will favor higher expression, and near saturation, it will favor lower expression.

The exceptional three cyanobacteria differ from other cyanobacteria carrying similar MTases in that their low level of HIP1 sequences, below the set point, could enable the ratchet mechanism to operate. Also, two of the exceptional strains, *Calothrix* PCC 7103 and *Cyanothece* PCC 7822, carry two copies of genes encoding the pertinent MTase, which may promote methylation of GC sites at the replication fork before mismatch repair takes place. However, the third strain, *Oscillatoria* PCC 10802, like most Group B1 cyanobacteria, carries only one copy of an rCCGGy-specific MTase. Perhaps a mutation increased the expression of the gene encoding the MTase. Or perhaps the expression of the MTase is truly lower than that of the MTases of the other two strains, and the slower accumulation of G^me^C may be related to the failure of the GCCGGC form of rCCGGy to reach fixation, as described in the previous section. That failure partially explains the anomalously high frequency of rCCGGy relative to other HIPs (Figure 2B). Other HIP sequences have G^me^C on both strands, but with rCCGGy, this is the case only for 32% of the instances (*i.e.*, only for GCCGGC). Also, the observed bias in sequences flanking rCCGGy (Table 3) may indicate a weak preference of the putative MutH analog in *Oscillatoria* PCC 10802 for a target somewhat more strict than G^me^C.

### 3.6. Why Do Symbiotic Cyanobacteria Lose HIP1?

Of the five obligate symbiotic cyanobacteria considered in this study, three have very low HIP1 O/E ratios, accompanied by low or very low CGATCG O/E ratios (strains **e**, **f** and **j** in Table 2 and Figure 5, columns 1, 11 and 12). A fourth has a HIP1 O/E amongst the lowest (strain **k**). Why should the putative benefits provided by overrepresented HIP1 sequences and perhaps G^me^C-dependent MMR not be of selective value to these strains?

The purpose of MMR is to correct mutation that arises during the course of DNA replication. This may be particularly important to phototrophic organisms such as cyanobacteria, because photosystem II (PSII) produces highly reactive singlet oxygen [42], the major contributor to photooxidative damage in plants [43]. The damage evidently extends to DNA, as the pattern of mutations in chloroplasts matches what is expected from the incorporation into nascent DNA of 8‑hydroxyguanine (also called 7,8-dihydro-8-oxoguanine) produced by singlet oxygen [44]. Phototrophs have defenses; incorporated 8-hydroxyguanine paired with adenine can be detected and corrected by MMR [45] as well as by other repair pathways [46]. One might therefore expect phototrophs to have highly active MMR systems (or the functional equivalent).

PSII and its oxidative challenges are present in almost all cyanobacteria, but two of the symbionts, *E. turgida* EtSB endosymbiont (**e** in Table 2) and UCYN-A (**f**) have lost PSII [16,18]. A third, *Richellia intracellularis HH01* (**j**), has some but not all genes of PSII, and transcription from the remaining PSII-encoding genes is very low [47]. Photosystem II is intact in a fourth symbiont, *Nostoc azollae* 0708, but the cyanobacterium exhibits little photosynthetic activity within its host plant [48]. These observations are reasonable, as all four symbionts evidently rely on their photosynthetic hosts for carbon while providing their hosts with fixed nitrogen. The fifth symbiont, *Prochloron didemni* P4-Papua New Guinea, lives within an animal host. This cyanobacterium has all the genes required for PSII, consistent with its role in providing its host with carbon through photosynthesis [17]. It is also the only symbiont considered in this study that has a level of HIP1 comparable to the levels exhibited by its close relatives.

The symbionts associated with plant hosts may have a reduced need for MMR (owing to their reduced or nonexistent reliance on PSII), hence (by hypothesis) a reduced need for G^me^C methylation. Also, if HIP1 functions as a target to initiate recombination or gene conversion as a means to repair photodamaged DNA, then perhaps the photosynthesis-deficient symbionts would be able to dispense with the sites. In this regard, it is interesting that a limited sampling of cyanobacteria indicates that Group B1 and B2 cyanobacteria tend to be polyploid, sometimes highly so, but Group C1 cyanobacteria (lacking HIP1 sites) tend to be monoploid [49] and therefore less likely to benefit from intergenome recombination.

## 4. Experimental Section

### 4.1. Phylogenetic Trees

Phylogenetic trees were made from sequences aligned using Clustal W [50] implemented through the CyanoBIKE instance of BioBIKE [10], an online integrated data/programming platform. For maximum likelihood trees, the most informative positions of the alignment were extracted using Gblocks [51] and then run through PhyML 3.0 [52], based on a GTR model and 4 substitution rate categories. Trees were visualized using FigTree 1.4.2 [53]. Alternatively, neighbor joining trees were constructed using PHYLIP [54] as implemented in BioBIKE.

### 4.2. Protein and Nucleotide Sequences

All DNA sequences were provided within BioBIKE from sequences deposited in GenBank. Protein sequences were also provided within BioBIKE except for the cases of six strains that were not annotated at the time they were uploaded into the database: *Chlorogloeopsis fritschii* PCC 6912, *Fischerella thermalis* PCC 7521, *Mastigocladopsis repens* PCC 10914, *Microchaete* PCC 7126, *Prochloron didemni* P4 Papua New Guinea, and *Scytonema hofmanni* UTEX 2349. For these strains, genes and pseudogenes were inferred on the basis of similarity between known protein and translated DNA sequences.

MTases and REases were identified in genomes by multiple means, including BLAST searches [55] and searching REBASE, a curated database of restriction and modification enzymes [33]. The integrity of MTase genes was assessed crudely by the length and *e*-value of BLAST matches and more carefully by inspection of protein sequence alignments, looking for the presence of conserved motifs of m5C MTases [20] and m6A and m4C MTases [56]. The target specificities of MTases were taken from REBASE, which draws its predictions from a partially automated process (enabled by SEQWARE) that considers overall sequence similarity to experimentally proven enzymes, the presence and positions of functional motifs, and similarity in the domains associated with target recognition [57,58]. Relying on protein families, such as Pfam [59], is not an effective strategy, as they do not distinguish between MTases related by sequence but unrelated by specificity. For example, the *Anabeana* PCC 7120 MTases All0061 (GATC) and Alr1052 (GGCC) are both members of Pfam:MethyltransfD12.

### 4.3. Calculation of Occurrences of Oligomer Sequences

Counts of oligomers over entire genomes were normalized to the total length of the genomes, using the COUNT-OF and LENGTH-OF functions of BioBIKE. The expected counts of an oligomer, used in the observed to expected ratio (O/E), was calculated from the nucleotide composition of the oligomer, using the formula:

(genome length) (GC/2)^G+C^ (0.5 − GC/2)^A+T^

where GC is the GC fraction of the genome (determined using BioBIKE's GC-FRACTION-OF function), and G, C, A, and T are the number of the specified nucleotides in the oligomer sequence. The counts and O/E ratios of all oligomers of a specific length were determined using the COUNTS-OF-K-MERS function of BioBIKE. Markov biases were calculated as previously described [39]. Counts of sequence patterns (e.g., rCCGGy) made use of BioBIKE's MATCHES-OF-PATTERN function.

To represent O/E ratios graphically, shades of red were assigned to O/E values greater than 1 and shades of green to O/E values less than 1. The shades were chosen according to a role that was applied uniformly to all genomes and all size classes of oligomer, using the binomial probability **P** for the frequency of an oligomer (calculated using a typical genome size), with extreme O/E values fixed as follows:

- Most deviant red: C_obs_/C_exp_ such that Log_10_(P_obs_/P_exp_) = 8000
- Least deviant red: C_obs_/C_exp_ such that Log_10_(P_obs_/P_exp_) = 100
- Least deviant green: C_obs_/C_exp_ = C_obs_/C_exp_ for least deviant red
- Most deviant green: C_obs_/C_exp_ = C_obs_/C_exp_ for most deviant red
- Intermediate shades distributed linearly between extreme shades

The extreme values of 100 and 8000 were chosen in order to produce a range of shades that distinguishes salient features of O/E ratios.

To reduce noise in the presentation of normalized counts, counts were filtered, letting pass only those with O/E ratios larger than a threshold C_obs_/C_exp_ value of 1000, calculated as described above.

## Supplementary Materials

Supplementary materials can be accessed at: http://www.mdpi.com/2075-1729/5/1/921/s1.
