# Supplementary material for: Highly Iterated Palindromic Sequences (HIPs) and Their Relationship to DNA Methyltransferases
Source: Life (Basel). 2015 Mar 17;5(1):921–48. doi: 10.3390/life5010921 (PMC4390886; doi:10.3390/life5010921)
Supplement: Supplementary file 1 [file life-05-00921-s001.zip › HIP-Suppl-Figures.pdf]

Supplementary Materials

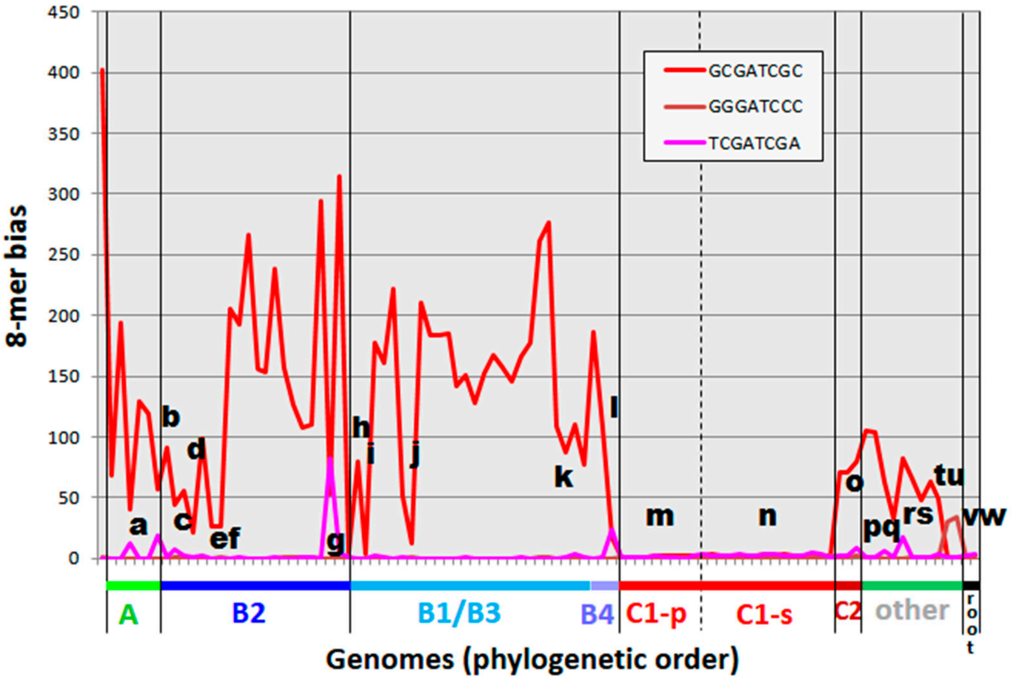

(a)

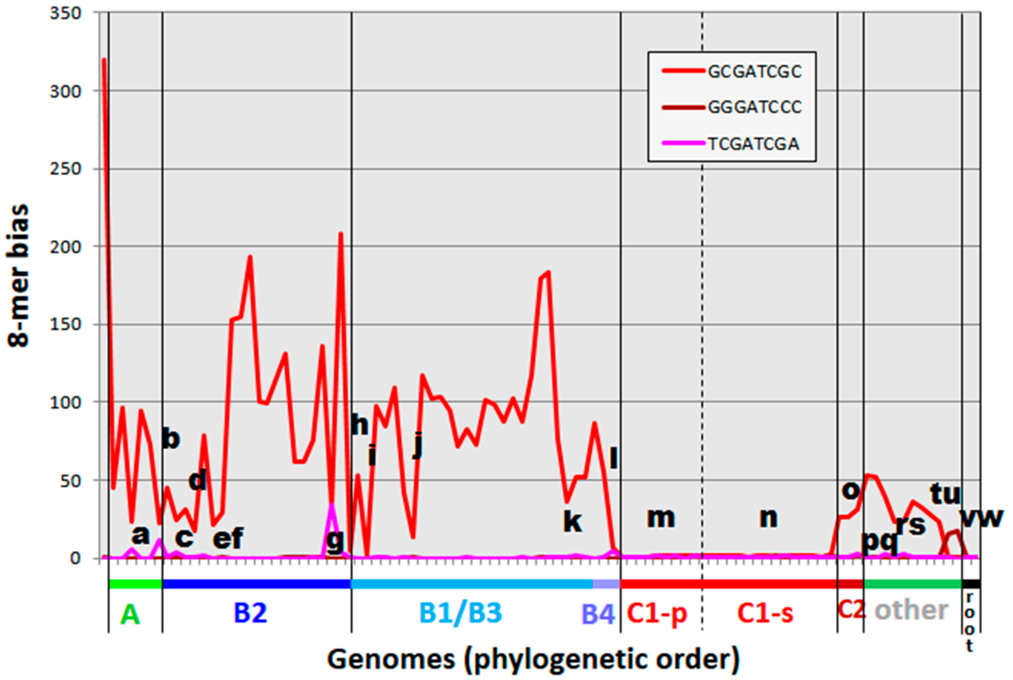

(b)

Figure S1. (A). Occurrences of HIP1 and other oligomers in cyano-bacterial genomes. Bias is calculated from a 1st-order Markov model, thereby taking into account mono- and dinucleotide frequencies. Some genomes of interest are marked with letters, explained in Table 2. Underlying numbers are given in Supplemental Table S2; (B) Occurrences of HIP1 and other oligomers in cyano-bacterial genomes. Bias is calculated from a 2nd-order Markov model, thereby taking into account mono-, di-, and trinucleotide frequencies. Some genomes of interest are marked with letters, explained in Table 2. Underlying numbers are given in Supplemental Table S2.

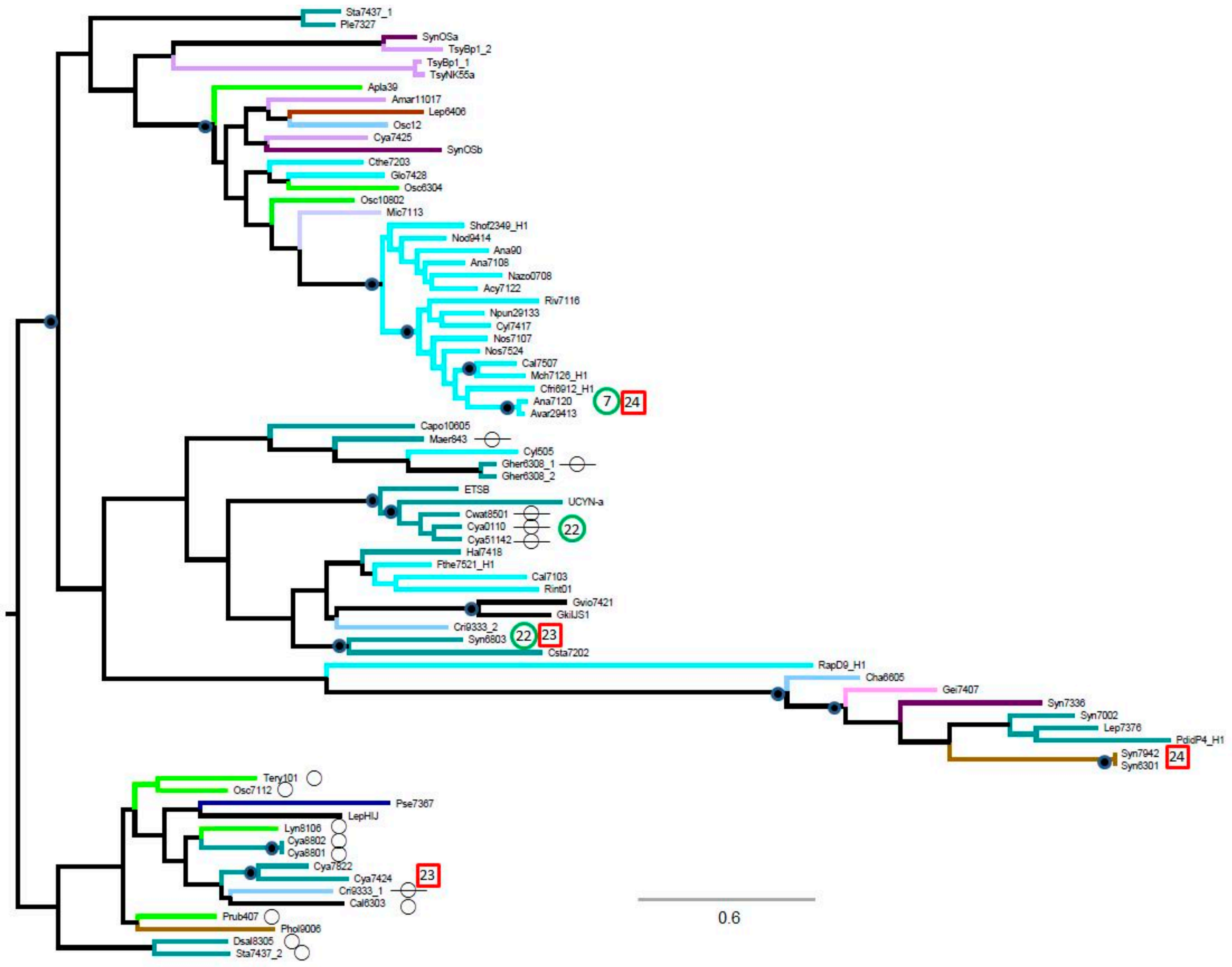

(**a**)

Figure S2. *Cont.*

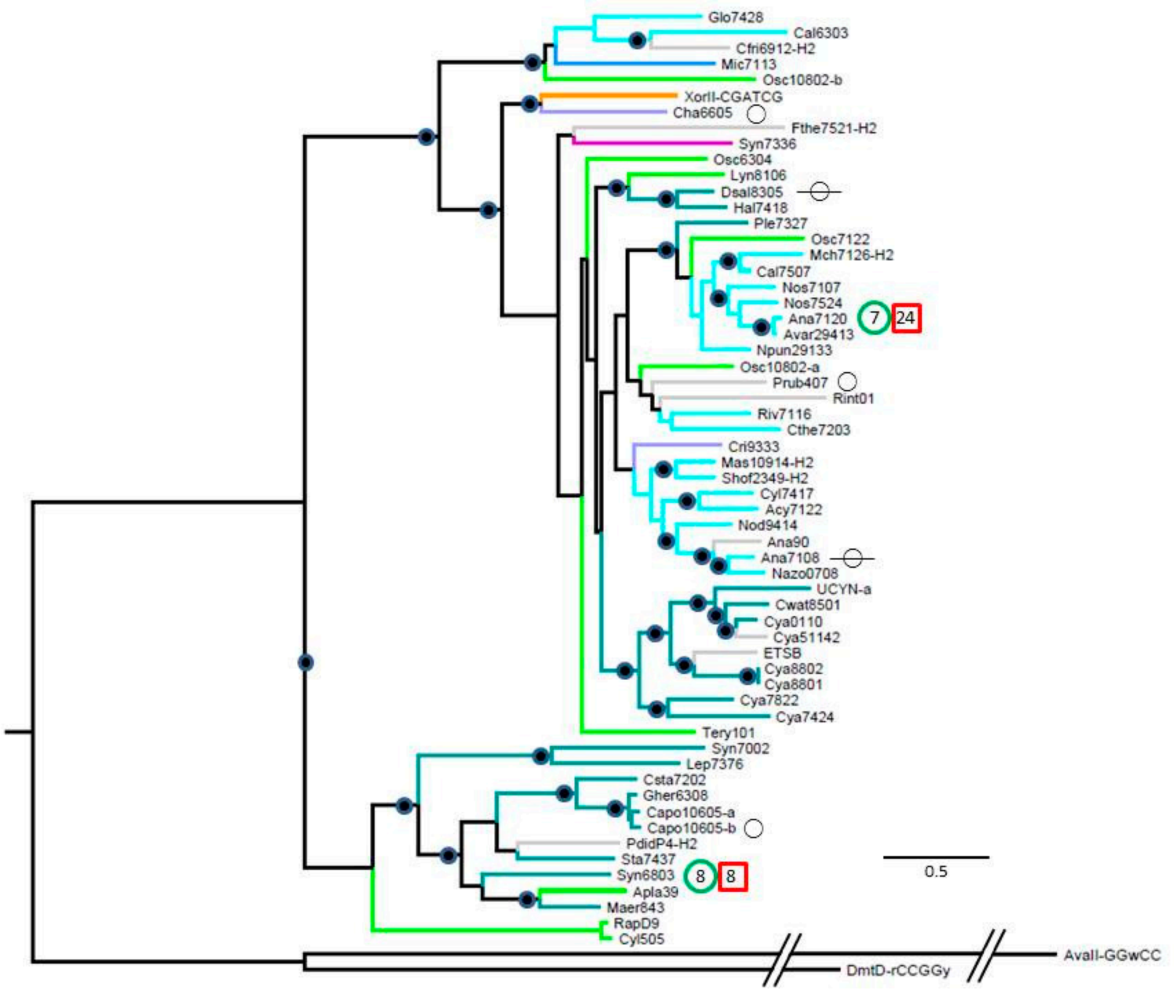

(**b**)

Figure S2. Maximum likelihood phylogenetic trees of methyltransferases. Conventions are as in Figure 1. In addition, open circles indicate the presence of a gene encoding a protein similar to a cognate REase. A line through the circle indicates that the gene is defective. Circled numbers are references to articles providing evidence for function of the MTase. Boxed numbers are references to articles providing evidence for methylation of the genome. (**A**) Maximum likelihood phylogenetic tree of m6A, group alpha, GATC-specific MTases, rooted by the m4C, group alpha GGCC-specific MTase DmtB from *Anabaena* PCC 7120. (**B**) Maximum likelihood phylogenetic tree of m5C CGATCG-specific MTases, rooted by the m5C rCCGy-specific MTase DmtD and GGwCC-specific MTase AvaII from *Anabaena* PCC 7120.
